# Ultrasound probe tilt impedes the needle-beam alignment during the ultrasound-guided procedures

**DOI:** 10.1038/s41598-021-81354-w

**Published:** 2021-01-15

**Authors:** Qingxiang Mao, Haitao He, Yuangang Lu, Yi Hu, Zhen Wang, Maoxiang Gan, Hong Yan, Liyong Chen

**Affiliations:** 1 Department of Anesthesiology, Daping Hospital, Army Medical University, 10 ChangjiangZhilu, Yuzhong District, Chongqing, 400042 China; 2 Department of Maxillofacial and Head and Neck Surgery, Daping Hospital, Army Medical University, Chongqing, China; 3 Department of Plastic and Cosmetic Surgery, Daping Hospital, Army Medical University, Chongqing, China

**Keywords:** Ultrasonography, Preclinical research

## Abstract

The objective of this study was to identify the factors that complicate the needle visualization in ultrasound-guided in-plane needling procedures. Forty-nine residents were recruited and randomized to insert the simulated blood vessel with four different views including Neutral (the long axis of the probe along the visual axis and the ultrasonic beam vertical to the surface of gel phantom), 45°-rotation (45° angle between the long axis of probe and the operator’s visual axis), 45°-tilt (45° angle between the ultrasonic beam and the surface of gel phantom) and 45°-rotation plus 45°-tilt of probe. Number of needle redirections, insertion time, and needle visibility were documented and compared for each procedure. When the residents faced with 45°-tilt view, the needle redirections (2 vs 0) and insertion time increased significantly (39 *vs* 16) compared with that of the Neutral view. When faced with 45°-rotation plus 45°-tilt view, the residents’ performance decreased further as compared with that of the 45°-tilt view and the Neutral view. However, there was no performance difference between the Neutral view and 45°-rotation view. In conclusion, during ultrasound-guided in-plane procedures, tilting the ultrasound probe may increase the difficulty of needle-beam alignment.

## Introduction

In ultrasound-guided regional anesthesia, the in-plane technique allows precise targeting of the nerve and real-time visualization of needle trajectory^1,2^. Nevertheless, failure to align needle and ultrasonic beam imposes the risk of accidentally damaging adjacent vital structures not visible on the ultrasound screen^3,4^. Tilting and rotating are common movements of probe and they help to optimize the visualization of the targeted nerves or vessels. During the ultrasound-guided supraclavicular brachial plexus block, for example, the transducer is always placed in an oblique transverse plane (rotation) and tilted to supraclavicular fossa (tilt)^5,6^. However whether these maneuvers themselves have any potential influences on needle-beam alignment is not known.

In the present study, we compared the resident volunteers’ performance of ultrasound-guided vessel insertion in four different standard scenarios including Neutral, 45°-rotation, 45°-tilt and 45°-rotation plus 45°-tilt of probe. We hypothesized that these manipulation maneuvers would increase the difficulty of needle-beam alignment and decrease the success rates.

## Methods

**Figure 1:**
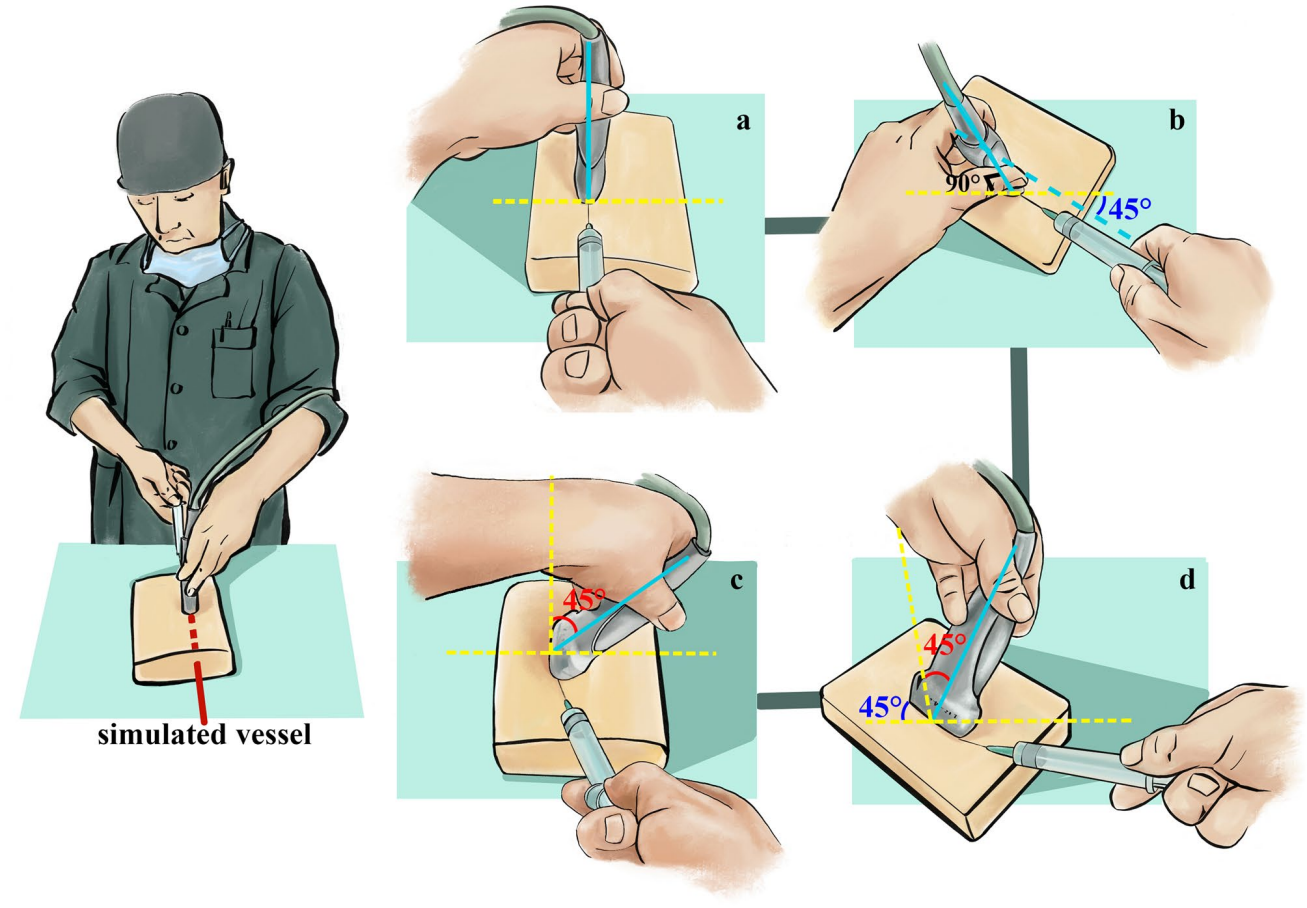
A schematic drawing of the four insertion views in phantom study. The simulated vessel is represented by the red dashed line. (**a**) Neutral view, the long axis of ultrasound probe was along the operator’s visual axis and ultrasonic beam was vertical to the surface of gel phantom; (**b**) 45°-rotation view, there is a 45° angle between the long axis of probe and the operator’s visual axis (or sagittal plane); (**c**) 45°-tilt view, there is a 45° angle between the ultrasonic beam and the vertical line (or the surface of gel phantom); (**d**) 45°-rotation plus 45°-tilt view, there is 45° angle between the long axis of probe and the operator’s visual axis (or sagittal plane), while there is another 45° angle between the ultrasonic beam and the vertical line. Yellow dashed line: Vertical/horizontal reference line; Blue solid line: direction of ultrasonic beam; Green angle: rotation angle; Red angle: tilt angle. The copyright holder of this image has been acknowledged and agreed to publish it under a CC BY open access license.

The study protocol was adhered to the Declaration of Helsinki and was approved by the Ethics Committee of the Daping Hospital (2018-45). Forty-nine Anesthesia residents were enrolled in the phantom study and the informed consent was obtained from all subjects. Before tasks, participants were asked to complete a questionnaire about experiences in ultrasound-guided procedure. For residents who have no experience were provided with a 10-min standardized ultrasound-guided in-plane insertion instruction. Participants were asked to insert the long-axis of simulated blood vessel on a gel phantom (Ningbo Liuyedao Medical Technology Co., Ltd, China) with four different views including Neutral (the long axis of the probe was along the visual axis and the ultrasonic beam was vertical to the surface of gel phantom), 45°-rotation (there was 45° angle between the long axis of probe and the operator’s visual axis), 45°-tilt (there was 45° angle between the ultrasonic beam and the surface of gel phantom) and 45°-rotation plus 45°-tilt of probe (Fig. 1a–d). A M9 ultrasound machine (Mindray, Shenzhen, China) equipped with a high-resolution 9.0-MHz linear probe was used. The order in which insertion was performed with each view was determined by a computer-generated random number list. Successful insertion of vessel was defined as aspiration of fluid from the simulated vessel. All attempts were recorded by a video camera and analyzed by an attending physician. After completion of the tasks, all participants were asked to rank the four views from easiest to the most difficult. The participants and analyst were blinded to the study objectives.

The performance outcome data are reported as medians with interquartile ranges (IQRs). Statistical analysis was conducted by Mann–Whitney U test or Friedman’s two-way test with post hoc analysis using Dunn-Bonferroni test to compare the performance outcome. Demographic characteristics of study participants are reported as mean ± SD. Statistical analysis was conducted by one-way ANOVA or chi-squared. The level of significance was set at *P* < 0.05.

## Results

When faced with Neutral view (Fig. 1a), the operators showed the best performance outcome (Table 1). When there was 45° angle between the long axis of probe and the operator’s visual axis (rotation), the operator showed similar performance outcome with that in Neutral view.

**Table 1:** Performance of residents in four cannulation views and self-assessment (n = 49). Data were described as Median (IQR) and number (percentage).

Variables	Neutral	45° rotation	45° tilt	45° rotation and 45°tilt
*Skin breaks (times)*				
All residents	1 (1–1)	1 (1–1)	1 (1–2)	3 (1–6)^a, b^
Level 1 (n = 15)	1 (1–2)	1 (1–2)	1 (1–2)	4 (1–6.5)
Level 2 (n = 16)	1 (1–1)	1 (1–1)	1 (1–3.3)	3 (1–6.3)
Level 3 (n = 18)	1 (1–1)^c^	1 (1–1)	1( 1–2.3)	2 (1–3.4)
*Needle redirections (times)*				
All residents	0 (0–1)^b^	0 (0–2) ^b^	2 (0–5)^a^	5 (2–9)^a, b^
Level 1 (n = 15)	1 (0–2.5)	0 (0–2)	3 (0–5)	4 (2–11)
Level 2 (n = 16)	0 (0–1.3)	0.5 (0–2.3)	3.5 (0–5.75)	8 (5–11)
Level 3 (n = 18)	0 (0–1)	0 (0–1.1)	1 (0–4.9)	4 (2–7.0)
*Insertion time (s)*				
All residents	16 (11–34)^b^	20 (11–32)^b^	39 (19–80)^a^	113 (44–220)^a, b^
Level 1 (n = 15)	29 (13–63)	21 (14–51.5)	62 (24.5–111.5)	105 (44.5–287)
Level 2 (n = 16)	22 (14.8–27.5)	20 (9.8–27.5)	47.5 (20.3–89.3)	137 (83.8–195.3)
Level 3 (n = 18)	11 (10–26.4) ^c^	19.5 (11.3–32.4)	35.5 (18.3–71.1)	78 (41–140.7)
*Needle visibility*				
All residents	0.6 (0.27–0.75)	0.53 (0.19–0.75)	0.22 (0.11–0.5)	0.06 (0.03–0.13)^a,b^
Level 1 (n = 15)	0.32 (0.21–0.77)	0.38 (0.17–0.80)	0.20 (0.08–0.26)	0.04 (0.02–0.08)
Level 2 (n = 16)	0.59 (0.28–0.75)	0.54 (0.34–0.62)	0.24 (0.18–0.34)	0.06 (0.03–0.10)
Level 3 (n = 18)	0.72 (0.52–0.76)	0.55 (0.21–0.83)	0.23 (0.11–0.55)	0.1 (0.05–0.14)
*Number of residents who rated as most difficult view (percentage)*				
All residents	1 (2%)	2 (4.1%)	9 (8.4%)	37 (75.5%)
Level 1 (n = 15)	1 (6.7%)	1 (6.7%)	3 (20%)	10 (66.7%)
Level 2 (n = 16)	0 (0%)	1 (6.3%)	3 (18.8%)	12 (75%)
Level 3 (n = 18)	0 (0%)	0 (0%)	3 (16.7%)	15 (83.3%)

^a^*P* < 0.05 compared with the Neutral group; ^b^*P* < 0.05 compared with the 45° tilt group; ^c^*P* < 0.05 compared with Level 1 subgroup. Insertion Time: from skin puncture to aspiration of fluid; Needle visibility: fraction of time the needle tip was visualized during the insertion; Level 1: no experience of ultrasound-guided procedures; Level 2: have performed more than one but less than forty cases of ultrasound-guided procedures; Level 3: have performed forty cases or more.

When faced with 45°-tilt view, the number of needle redirections(2 vs 0, *P* = 0.012; 2 vs 0, *P* = 0.037) and insertion time (39 vs 16, *P* = 0.003; 39 vs 20, *P* = 0.003) increased significantly as compared with the Neutral and the 45°-rotation group.

When faced with the 45°-rotation plus 45°-tilt view, the operators unsurprisingly showed the worst performance outcome (Table 1). The number of skin breaks, needle redirections and the insertion time increased significantly, and needle visibility (fraction of time the needle tip was visualized during the insertion) decreased significantly, as compared with the other three groups. This indicates that although 45° rotation of probe alone will not impede the alignment, it will further increase the difficulty of needle-beam alignment when coexisted with 45°-tilt.

The self-assessment results showed that among the four different views, the 45°-rotation plus 45°-tilt view was rated as the most difficult by 75.5% residents (Table 1). These self-assessment results were consistent with the residents’ performance result.

According to prior experiences of ultrasound-guided procedures (Table 2), we divided the residents into three groups including Level 1 (no experience), Level 2 (have performed more than one but less than forty cases) and Level 3 (have performed forty cases or more). A subgroup analysis on the residents’ performance found that only in the Neutral view, the Level 3 residents showed less needle redirections (*P* = 0.011) and shorter insertion time (*P* = 0.028) when compared with Level 1 residents (Table 1). In other three insertion views, there were no significant differences in the residents’ performances between all three subgroups (Table 1).

**Table 2:** Demographic variable of participating residents.

	n (%)
*Gender*	
Male	25 (51.0)
Female	24 (49.0)
*Prior experience of ultrasound usage*^*a*^* (times)*	
0	15 (30.6)
1–39	16 (32.7)
≥ 40	18 (36.7)

^a^Prior experience of ultrasound usage includes ultrasound-guided nerve blocks or central venous catheterization.

## Discussion

It is recommended to utilize an in-plane technique for ultrasound-guided procedures because the entire needle and the depth of the needle tip can be visualized on the ultrasound image^2,7^. However ultrasound might give the inexperienced operators a false sense of security and mislead them to neglect traditionally taught principles with regard to needle direction. Blind advancement of the needle can lead to accidental damage of vital structures like artery or pleura^8,9^. It is important to identify the potential factors that may complicate the alignment of needle with ultrasound beam. These factors can be used to improve the clinical relevance of simulation training and quantitatively assess the difficulty of ultrasound-guided procedures.

Tilting and rotating are often used to optimize the visualization of the targeted nerves and bring the “disappeared” needle tip back^10,11^. In this phantom study, we found that the tilted probe will increase the difficulty of the needle-beam alignment. When the operators were faced with both rotation and tilt angles, the alignment is further complicated. The possible reason may be that the operators were less willing to tilt their head or body to keep the line of sight parallel to the ultrasonic beam plane. It is recommended that the operator should have the insertion site, the needle, and the ultrasound screen in the line of sight during needle insertion^2,10^. Our study indicates that if a probe tilt angle exists, the operator shall tilt their body or head to keep the line of sight parallel to the ultrasonic beam plane, in order to facilitate the needle-beam alignment.

Although in our study the 45° rotation of probe did not lead to a decreased performance, another phantom study found that needle advancement along the visual axis resulted in improved needle imaging and a shorter time to targeting when compared with that across the visual axis (90° rotation of probe)^12^. It is advisable for the operators to move their standing position to eliminate the rotation angle of probe.

Previous experiences of operator are presumed to be helpful to needle-beam alignment. However in our study, the level 3 residents who had performed more than 40 cases of ultrasound-guided procedures only showed better performance in the Neutral view while not did in 45°-rotation and/or 45°-tilt views. However, the sample size of subgroup was not large enough to make a reliable conclusion. The difference between phantom research and clinical practice may also complicate the extension of these results which warrants a well-designed clinical research.

In conclusion, our study found that both 45°-tilt and 45°-rotation plus 45°-tilt of probe would increase the difficulty of needle-beam alignment during in-plane procedures. Operators with experiences of ultrasound-guided procedures might also be affected by these probe manipulation angles. It is advisable for the operators to move their standing position and tilting their body or head to keep the line of sight parallel to the ultrasonic beam plane, in order to facilitate the needle-beam alignment.
